# Supplementary material for: NADP-Dependent Malic Enzyme Genes in Sweet Pepper Fruits: Involvement in Ripening and Modulation by Nitric Oxide (NO)
Source: Plants (Basel). 2023 Jun 17;12(12):2353. doi: 10.3390/plants12122353 (PMC10304560; doi:10.3390/plants12122353)
Supplement: Supplementary file 1 [file plants-12-02353-s001.zip › plants-2431272-supplementary.pdf]

```
NADP-ME1      MFSLNGTTFTDNSLCGISRCLTTQSRKRVSVPMVV--VSSNGRAADGNVSVLMENTLKES      58
NADP-ME5      MFSLNGSSFTNNSLSGVSRCLTQSC-RRVSAPMVVAAVSSNGKPGDGHVSVLVENALTES      59
NADP-ME3      -------------------------MISFTIPNFLRKALGKRRSGRINIRVVMESTLKEL      35
NADP-ME2      ------------------------------------------------------------      0
NADP-ME4      ----------------------------------------------------MESKMKEK      8

NADP-ME1      ---VAVIEKDDFKSTVSGGVGDVYGEDTASEDQTITPWTFSVASGYSLLRDPHYNKGLAF      115
NADP-ME5      ---PVPVEK-ETKSTVTGGVGDVYGEDSATEDQSITPWTLSVASGFSLLRNPHYNKGLAF      115
NADP-ME3      SNGESVLDV-KDKCGVGGGVEDLYGEDRATEDQTITPWTFSVASGYSLLRDPHYNKGLAF      94
NADP-ME2      ------MESTLKEQIPAGGVEDVYGEDCATEDQCITPWTIAVSSGYNLLRDPRYNKGLAF      54
NADP-ME4      S--ESVVDMSPTSAVVEGGVEDIYGEDCATEDQLITPWTFQVSSGYNLLRDPRYNKGLAF      66
                   ::     .     *** *:**** *:*** *****: *:**:.***:*:*******
                  Putative nitration
NADP-ME1      SEKERDSHYLSGLLPPVVVDQELQVKKVMNVLRQYDVPLQRYMAMMDLQERNERLFYKLL      175
NADP-ME5      SERERDTHYLRGLLPPVVITHDLQVKKMMNSIRKYDVPLQRYMAMMDLQEMNERLFYKLL      175
NADP-ME3      TDAERDAHYLRGLLPPSVSNQELQEKKVMHNLRLYQVPLQRYMAMMDLQERNERLFYKLL      154
NADP-ME2      TERERDAHYLRGLLPPVVSTQELQEKKLMQSIRQYDLPLHKYVAMMELEERNERLFYKLL      114
NADP-ME4      TEQERDVHYLRGILPPAVMPQELQEKRLMQTLRRYEVPLNKYVALMELQERNERLFYKLL      126
              :: *** *** *:*** *   :** *::*: :* *::**::*:*:*:*:* *********
                    NADP-binding site
NADP-ME1      MENVEELLPIVYTPTVGEACQKYGSIFKRPQGLFISLKEKGKILEVLKNWPEKKIQVIVV      235
NADP-ME5      IDNVEELLPIVYTPTVGEACQKYGSIFRRPQGLFISLKEKGKIHEVLKNWPEKKIQVIVV      235
NADP-ME3      IDNVEELLPVVYTPTVGEACQKYGSIFKRPQGLFISLKEKGKILEVLKNWPEKSIQVIVV      214
NADP-ME2      IDNVEELLPIVYTPTVGEACQKYGSLFKRPQGLYISLKEKGRILEVLKNWPERSIQVIVV      174
NADP-ME4      IDNVEELLPIVYTPTVGEACQKYGSIFRRPQGLYISLKEKGRILEVLKNWPERKIQVIVI      186
              ::*******:***************:*:*****:*******:* ********:.*****:
                  NADP-binding site
NADP-ME1      TDGERILGLGDLGCQGMGIPVGKLSLYTALGGIRPSACLPVTIDVGTNNEKLLEDEFYIG      295
NADP-ME5      TDGERILGLGDLGCQGMGIPVGKLSLYTALGGIRPSACLPVTIDVGTNNMNLLNDEFYIG      295
NADP-ME3      TDGERILGLGDLGCQGMGIPVGKLSLYTALGGVRPSVCLPITIDVGTNNQQLLDDEFYIG      274
NADP-ME2      TDGERILGLGDLGCQGMGIPVGKLALYTALGGVRPSACLPITIDVGTNNEKLLNDEFYIG      234
NADP-ME4      TDGERILGLGDLGCQGMGIPVGKLALYTALGGVRPSACLPITIDVGTNNEQLLNDEFYIG      246
              **********************:*******:***.***:********  :**:******
                                                 Metal-binding residues
NADP-ME1      LRQRRARGQEYAELLDEFMSSVKQTYGEKVLIQFEDFANHNAFDLLEKYGTSHLVFNDDI      355
NADP-ME5      LRQRRATGQEYAELLDEFMYAVKQNYGEKVLIQFEDFANHNAFDLLAKYGTSHLVFNDDI      355
NADP-ME3      LKQKRARGQEYAELLLEFMSAVKQNYGERVLIQFEDFANHNAFELLAKYRTTHLVFNDDI      334
NADP-ME2      LRQNRATGQEYYDFLHEFMSAVKQNYGEKILIQFEDFANHNAFELLAKYRTTHLVFNDDI      294
NADP-ME4      LRQKRATGKEYHDFLDEFMKAVKQNYGEKVLVQYEDFGNHNAFELLAKYGSTHLVFNDDI      306
              *:*.** *:** ::* *** :***.***::*:*:***.*****:** ** ::********

NADP-ME1      QGTASVVLAGLMAALNLVGGNLAQHTFLFLGAGEAGTGIAELIALEMSK-----QTGAPL      410
NADP-ME5      QGTASVVLAGLMAALNLVGGTLAEHTFLFLGAGEAGTGIAELIALEMSKQLFLVQTGIPL      415
NADP-ME3      QGTASVVLAGLIASLKLLGGTLADHTFLFLGAGEAGTGIAELIALEITK-----KTSVPL      389
NADP-ME2      QGTASVVLAGLIASLKLLGGALCDHTFLFLGAGEAGTGIAELIALEISK-----KTNTPV      349
NADP-ME4      QGTAAVVLAGLVASLKLLGGSLADHTFLFFGAGEAGTGIAELIALAISK-----KTNAPV      361
              ****:******:*:*:*:** *.:*****:**************** ::*     :*. *:

NADP-ME1      EETRKKIWMVDSKGLIVRSRMESLQHFKMPWAHDHEPVKELVNAVKLIKPTILIGSSGTG      470
NADP-ME5      EETRKKIWMVDSKGLIVKSRMEMLQHFKRPWAHDHEPVKELVNAVKSIKPTVLIGSSGAG      475
NADP-ME3      EVARKKIWLVDSKGLIVSSRVESLQHFKKPWAHDHEPVKELIDAVKAIKPTVLIGTSGVG      449
NADP-ME2      EETRRKIWLVDSKGLIVSGRKGTLQAFKKPWAHEHEPVNNLLDAVKAVKPSVLIGTSGVG      409
NADP-ME4      EEARKNIWLVDSKGLIVSSRKESLQAHKKPWAHEHEPVNNLLDTVKAIKPTAIIGTSGVG      421
              * :*::**:******** .*   ** .* ****:****::*:::** :**: :**:**.*

NADP-ME1      KTFTKEVVRTIATFNKFVMQKPVIFALSNPTSQSECTAEEAYTWSDGRAIFASGSPFAPV      530
NADP-ME5      RTFTKEVVQAMATFN----EKPIIFALSNPTSQSECTAEEAYSWSEGRAIFASGSPFAPV      531
NADP-ME3      KTFTKEVVEAMASLN----PKPLVMALSNPTSQAECTAEEAYTWSKGHAIFASGSPFDPF      505
NADP-ME2      RTFTKEVVEAMASMN----ERPLIMALSNPTSQAECTAEEAYTWSEGRAVFASGSPFPSF      465
NADP-ME4      RTFTKEVIEAMASIN----KRPLIMALSNPTEQSECTAEEAYAWSEGRAVFASGSPFPPV      477
              :******:.::*::*     :*:::******.*:********:**.*:*:*******  .

NADP-ME1      EYEGKVFMSGQANNAYIFPGLGLGLIISGAIRVHDDMLLAASEALAAEVSKENFEKGLIY      590
NADP-ME5      EYKGKVYASGQANNAYIFPGFGLGLIISGAIRVHDDMLLVASEALAEQVSQENFEKGLIY      591
NADP-ME3      EFEGRTFVSGQANNAYIFPGFGLGIIMSGTIRVHDDMLLAASEALAAEVTEDDYLKGRIY      565
NADP-ME2      EYNEKLYFPGQANNCYIFPGFGFGLVMSGTIRVHDDMLLAASEALAAQVTDEHYAKGMIY      525
NADP-ME4      EYNNKLHIPSQANNCYIFPGFGFGLVMAGAIRVHNDMLLAASEALAGQVTEEHYVKGIIY      537
              *:: : .  .****.*****:*:*::::*:****:****.****** :*:.:.: ** **

NADP-ME1      PPFANIRKISAHIAAKVAAKAYELGLATRLPQPENLVSYAESCMYSPSYRSYR      643
NADP-ME5      PPFANIRKISAHIAAKVAAKAYELGLATRLPEPKDLIAYAESCMYSPAYRSYR      644
NADP-ME3      PPFTNIRKISAHIAAEVAAKAYELGLATHLPRPKDLVKYAESCMYTPVYRHYR      618
NADP-ME2      PPFADIRKISAHIAARVAAKAYELGVATRLPRPADLVKYAESCMYTPNYRSYR      578
NADP-ME4      PPFGIIRKISAHIAANVAARAYGLGVATRLPQPADLVKYAESCFYSPNYRCYR      590
              ***  **********.***:** **:**:**.* :*: *****:*:* ** **
```

Supplementary Figure S1. Alignment of the five NADP-ME sequences from pepper identifying the conserved NADP (blue) and metal binding (green) sites, and the possible tyrosine (Y) residue capable of being nitrated (orange). Asterisks denote the shared amino acids among all NADP-MEs.
